# New key management scheme lattice-based for clustered wireless sensor networks

**DOI:** 10.1371/journal.pone.0290323

**Published:** 2023-08-30

**Authors:** Jiang Zhang, Qi Liu

**Affiliations:** The Second Peoples Hospital of Jingdezhen, Jingdezhen, China; University of the West of Scotland, UNITED KINGDOM

## Abstract

Aiming at the quantum algorithm which can solve the problem of large integer decomposition and discrete logarithm in polynomial time, an anti-quantum computing key management scheme for clustered sensor networks is proposed in this paper. The lattice-based cryptosystem is used to achieve the anti-quantum performance of the key management scheme, and the security of the network is further improved through the mutual authentication of sensor network nodes. Due to the limited storage space of sensor nodes, this paper adopts the cluster management of wireless sensor networks, and most sensor nodes only need a small amount of storage space, thus reducing the deployment cost. Cluster management is suitable for medium and large-scale deployment of sensor networks. Because the data traffic is much larger than that of mutual authentication, the sensor nodes in wireless sensor networks use symmetric keys to communicate with each other after mutual authentication, which can effectively improve the communication efficiency in the case of frequent data communication. Experiments show that the authentication scheme based on lattice cryptosystem proposed in this paper will not improve with the continuous improvement of the security level, and its authentication scale will maintain a relatively stable state, while the algorithm scheme based on RSA will increase the authentication cost with the continuous improvement of the security level, so the scheme proposed in this paper is more suitable for application in the environment with high security level. This scheme can effectively reduce the cost of mutual authentication of sensor nodes, is conducive to the expansion of the network, and can ensure the security of authentication between sensor nodes even in the post-quantum era.

## 1. Introduction

Wireless sensor network integrates micro-electric technology, sensor technology and communication technology, and can be widely used in education, military, medical, transportation and other fields [1–5]. The security problems of wireless sensor networks come from the characteristics of wireless communication, the strict limitation of sensor node resources and the extensive and dense distribution area of sensor networks. Therefore, it is urgent to ensure the security of wireless sensor networks [6–9]. In 2016, Mehmood et al. A secure inter-cluster multi-key distribution scheme for wireless sensor networks is proposed [10]. In 2017, Zhang et al. A key establishment scheme for wireless sensor networks based on polynomial and random key pre-distribution scheme is proposed [11]. In 2022, Kumar et al. The cryptanalysis and improvement of mutual authentication protocol for real-time data access in industrial wireless sensor networks are proposed [12]. At the end of last century, Shor proposed a quantum algorithm to solve the problem of large integer decomposition and discrete logarithm in polynomial time, which made the research of cryptosystem against quantum computing attacks received great attention. At present, the development of special quantum computer is very rapid, and the solving time of traditional mathematical difficult problems such as large integer decomposition and discrete logarithm has reached the order of minutes, which brings a great threat to the classical encryption algorithms based on this kind of difficult problems. Post-quantum cryptography algorithm plays an important role in the security protection of user information in distributed systems in the quantum era when general quantum computers are widely used in the future. Among them, lattice cryptosystem has been concerned and studied by many scholars in recent years because of its advantages in efficiency and security [13–18]. Lattice cipher is a more practical post-quantum cryptographic algorithm [19–22].

### 1.1. Related work

In 2018, Mehmood et al. proposed a novel secure session key establishment scheme for wireless body area networks in the medical field. In the proposed scheme, in order to address the important issues of security and patient information privacy in wireless body area networks in medical applications, session keys are established for a specific period of time in order to securely communicate information related to patient health vital signs. Important data, ensuring the security and privacy of vital signs related to the human body [23]. In 2019, Bootle et al. proposed the algebraic techniques for short exact lattice-based zero-knowledge argument of knowledge systems [24]. In 2020, Mehmood et al. proposed an energy-efficient and reliable trust-based communication scheme for remote patient monitoring in wireless body-area networks, where trust and privacy-preserving enforcement is critical as important parameters are communicated to remote locations. In WBAN, trust among stakeholders is very important and is considered as a critical success factor for the reliability of information exchange between them [25]. In 2021, Lyubashevsky et al. proposed a shorter lattice-based zero-knowledge argument of knowledge systems via one-time commitments [26]. In 2021, Mehmood et al. proposed an efficient and secure session key management scheme for wireless sensor networks is proposed. In the proposed scheme, the main steps of public-key encryption in asymmetric cryptosystems are minimized, and most public-key encryption operations are based on symmetric-key encryption. This solution can greatly reduce the energy consumption of the wireless sensor network and ensure better security [27]. In 2022, Mehmood et al. proposed a Mobile Agent-Based Energy-Efficient Data Aggregation Scheme for Wireless Body Area Networks. Among the proposed schemes, reliable data aggregation in WBAN is very important to ensure data delivery as soon as possible in healthcare applications. This scheme solves the shortcoming of client-server sending data, and the mechanism of mobile agent proposed in this scheme proves to be a more feasible solution [28]. In 2023, Dharminder et al. [29] an efficient lattice-based authenticated key exchange protocol using a ring-based learning assumption with errors is designed for IoT smart devices, which is robust to different attacks.

In practical applications, most of the existing wireless sensor network key management schemes are based on traditional cryptographic systems such as large integer decomposition and discrete logarithm problems. In the quantum era when general-purpose quantum computers are popularized in the future, these algorithms will pose a huge threat. Therefore, it is necessary to study how to combine sensor network technology with anti-quantum attack technology, so that wireless sensor networks have the security against quantum computing attacks, and design and optimize the deployment scheme of sensor nodes according to the application scenarios of wireless sensor networks to reduce the deployment time. cost and improve communication efficiency. This paper studies the security protocol based on lattice cryptography, which can provide ideas for the security of wireless sensor networks and better protect the privacy data security of wireless sensor networks.

### 1.2. Overview of the paper

In practical applications, most of the existing key management is based on traditional cryptosystems such as large integer decomposition and discrete logarithm problems. In the future quantum era of universal quantum computers, these algorithms will pose a great threat. Therefore, it is necessary to research and develop a negotiation method and system based on key update in the post-quantum era. The rest of the paper is organized as follows: in Section 2, we introduce some basic concepts and algorithms of lattice schemes. In Section 3, we give our network model. In Section 4, we proposed a key management scheme. In Section 5, we analyze the correctness, security. In Section 6, finally, we summarize the key management scheme.

## 2. Preliminaries

**Definition 2.1**. ([30]). Let Λ be an *n*-dimensional lattice and *ε*>0. Then, the smoothing parameter *η*_*ε*_(Λ) is the smallest real *s*>0 such that $\rho_{\frac{1}{\sqrt{2\pi }s}}(\Lambda^{*}\backslash \{0\})\leq \varepsilon$.

**Lemma 2.2** ([30]). For any *n*-dimensional lattice Λ with basis *B* and *ε*>0, we have:

$$\eta_{\varepsilon }(\Lambda )\leq \|\tilde{B}\|\cdot \sqrt{\ln(2n/(1+1/\varepsilon ))/\pi }$$

**Lemma 2.3** ([31]). Let *m*,*k*>1, Λ be *m*-dimensional lattice and *c*∈*Z*^*m*^. Then:

$${\mathrm{Pr}}_{Z}\leftarrow D_{\sigma }[|z|>k\sigma ]\leq 2e^{\frac{-k^{2}}{2}}$$

$${\mathrm{Pr}}_{Z}\leftarrow D_{\sigma }^{m}[{\left\|z\right\|}_{2}>k\sigma \sqrt{m}]\leq k^{m}e^{\frac{m}{2}(1-k^{2})}$$

$${\mathrm{Pr}}_{Z}\leftarrow D_{\Lambda ,\sigma ,c}^{m}[{\left\|z\right\|}_{2}>k\sigma \sqrt{m}]\leq 2k^{m}e^{\frac{m}{2}(1-k^{2})}$$

**Lemma 2.4** ([31]). Let *Q*∈*Z*^*m*×*n*^ and Λ be an *n*-dimensional lattice. Then, for any $\sigma \in R_{>0}^{m}$ and *s*∈*R*^*m*^ we have:

$$\frac{\rho_{\sigma }(s)}{\sum_{z\in \Lambda }\rho_{\sigma }(Qz)}\leq \frac{\rho_{\sigma }(s)}{\sum_{z\in \Lambda }\rho_{\sigma }(s+Qz)}\leq \frac{1}{\sum_{z\in \Lambda }\rho_{\sigma }(Qz)}$$

**Theorem 2.5** ([32]). Let *A*∈*Z*^*n*×*m*^ and *W*∈*Z*^*k*×*m*^ be arbitrary matrices and denote *w*_*i*_∈*Z*^*m*^ to be the *i*-th row of *W*. Furthermore, suppose *σ* = (*σ*_1_,*σ*_2_,⋯,*σ*_*k*_) satisfies for $\sigma_{i}\geq q^{n/m}\sqrt{\frac{ek}{m}}\|w_{i}\|+2$. Then, for any *s*∈*R*^*k*^, we have:

$$\frac{\rho_{\sigma }(s)}{\sum_{z\in \Lambda_{q}^{⊥}(A)}\rho_{\sigma }(s+Wz)}\leq \frac{1}{2}$$

**Definition 2.6** Module-SIS(MSIS_*n*,*m*,*B*_) ([31]). Given $A\leftarrow R_{q}^{n\times m}$, the Module-SIS problem with parameters *n*,*m*>0 and 0<*B*<*q* asks to find $z\in R_{q}^{m}$ such that *Az* = 0 over *R*_*q*_ and 0<‖*z*‖<*B*. An algorithm *ψ* is said to have advantages *ϵ* in solving MSIS_*n*,*m*,*B*_ if:

$$\mathrm{Pr}[0<\|z\|<B\wedge Az=0|A\leftarrow R_{q}^{n\times m};z\leftarrow \psi (A)]\geq \epsilon$$

**Definition 2.7** (MLWE_*n*,*m*,*χ*_) ([32]). Given $A\leftarrow R_{q}^{n\times m}$, a secret vector *s*←*χ*^*m*^ and error vector *e*←*χ*^*n*^, the Module-LWE problem with parameters *n*,*m*>0 and an error distribution *χ* over *R* asks the adversary *ψ* to distinguish between the following the cases: (*A*,*As*+*e*) for *A*. Then, *ψ* is said to have advantages *ϵ* in solving MLWE_*n*,*m*,*χ*_ if

$$\left|\mathrm{Pr}[b=1|A\leftarrow R_{q}^{n\times m};s\leftarrow \chi^{m};e\leftarrow \chi^{n};b\leftarrow \psi (A,As+e)\right]$$

$$-\mathrm{Pr}[b=1|A\leftarrow R_{q}^{n\times m};b\leftarrow R_{q}^{n};b\leftarrow \psi (A,b)]|\geq \epsilon$$

**Lemma 2.8** (Lattice Trapdoors [30]). TrapSamp(1^*n*^,1^*m*^,*q*). That, given any integers *n*≥1, *q*≥2, and sufficiently large *m* = Ω(*n*log*q*), outputs a matrix $A\in Z_{q}^{n\times m}$ and a trapdoor matrix *T*∈*Z*^*n*×*m*^ such that the distribution of *A* is *negl*(*n*)-close to uniform.

**Lemma 2.9** ([33]). Let *n*, *p* be positive integers. Let *Λ* be a lattice of rank *n*, and let *V* = [−*p*,*p*]^*n*^. Let $T=p\sqrt{5n(1+\delta )/8}$, where

$$\delta =\sqrt{\frac{32(\lambda +1)}{25n\log_{2}e}}.$$

Define h the distribution obtained by sampling *α* from [−*p*,*p*] and *s* from $\psi_{1}^{n}$ and outputting *v* = *α*⋅*s*. Further, let *M*>1, $t=\sqrt{\left(\lambda +2)/(\pi \log_{2}e\right)}$ and definitely

$$\sigma_{\min}={\left(-t+\sqrt{\frac{t^{2}+\ln(M)}{\pi }}\right)}^{-1}\cdot T.$$

Let *σ*≥*σ*_min_. We now define two distributions *P*_1_: Sample *v*←*h* and *y*←*D*_Λ,*σ*_. Define *z* = **y** + *v*. Output (*v*, *z*) with probability

$$\min(1,\frac{D_{\Lambda ,\sigma }(z)}{M\cdot D_{\Lambda ,\sigma }(z-v)}).$$

*P*_2_: Sample *v*←*h* and *z*←*D*_Λ,*σ*_. Output (*v*, *z*) with probability 1/*M*.

Then, it holds that *P*_1_ outputs something with probability at least (1−2^−*λ*^)/*M*, and that

$$\Delta (P_{1},P_{2})\leq 2^{-(\lambda +1)}(1+1/M)\leq 2^{-\lambda }.$$

## 3. Network model

**Fig 1:**
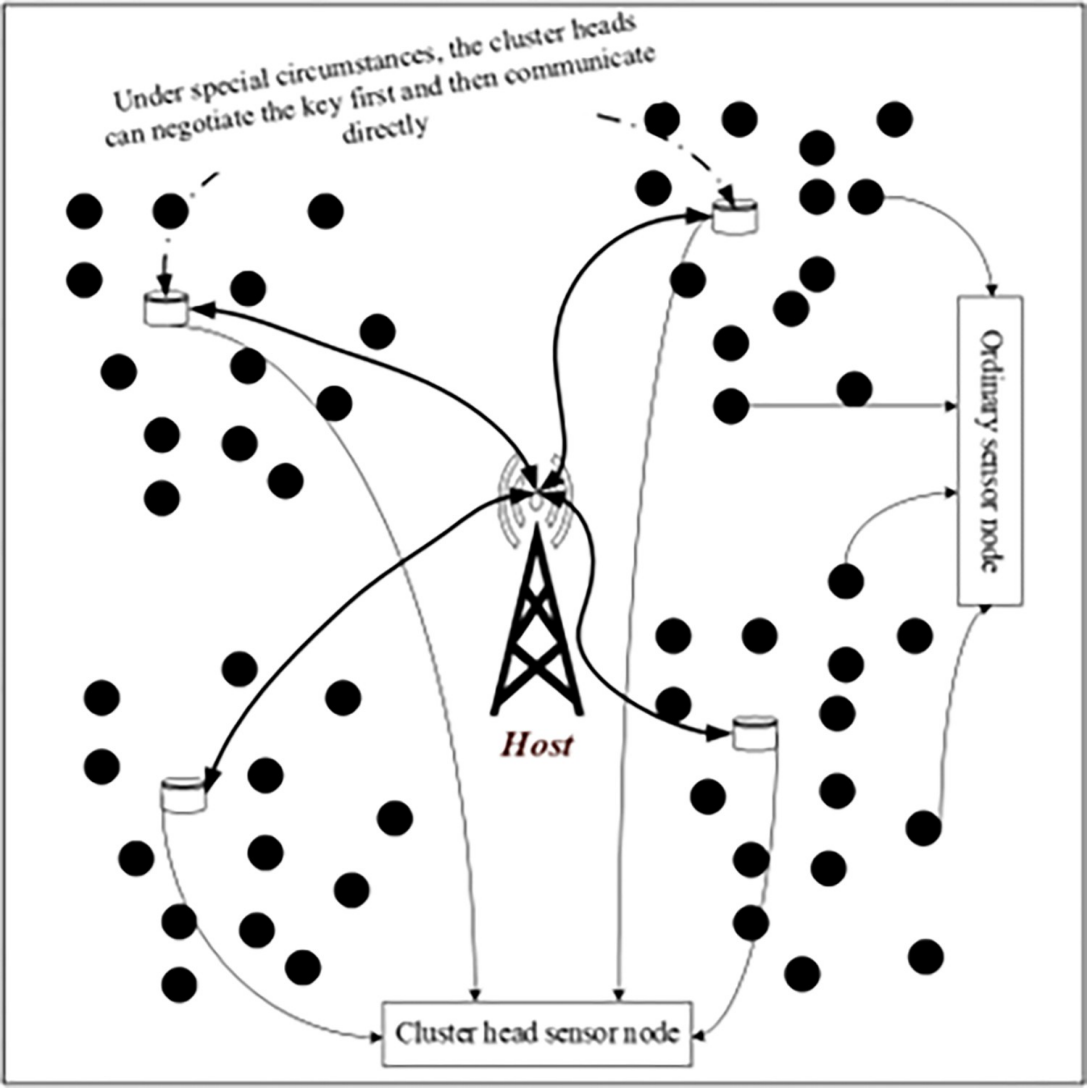
Wireless sensor network model.

Wireless sensor networks generally have two kinds of topologies: planar structure and hierarchical structure. All the nodes in the flat structure network are equal, and there is no bottleneck in principle, so it is relatively robust. However, its biggest disadvantage is that the network size is limited, the routing maintenance cost is high, and the energy consumption is relatively high. In the hierarchical structure, the network is divided into clusters, and each cluster is composed of a cluster head node and multiple cluster members, so it is also called heterogeneous network. Cluster head nodes form a higher-level network, which is responsible for the collection and forwarding of data between clusters. The use of cluster structure can reduce the energy cost caused by transmission and is conducive to network expansion. In this method, the clustered wireless sensor network model will be used to manage the key of the sensor nodes. The sensor network model is shown in Fig 1.

In this paper, an anti-quantum key management method for clustered sensor networks is proposed. The details include:

1. This paper assumes that each cluster head sensor node is assigned its own identity and a pair of public and private keys based on lattice public key cryptosystem. The cluster head sensor node plays a key role in the network, which can communicate directly with the host, collect the information sent by the ordinary sensor node in the cluster and forward it to the host.
2. All sensor nodes in each cluster communicate securely through the symmetric key shared by the cluster, and the ordinary sensor nodes in the cluster can communicate directly. Indirect communication can be carried out through the cluster head and the host or sensor node outside the cluster.
3. When the cluster head sensor node finds that the communication key is not secure, it should deal with it in time and redistribute the new communication key through the host.
4. In special cases, the cluster head sensor node can also communicate directly by negotiating the communication key. In order to further enhance the security of the symmetric key used for communication, the aging of the symmetric key can be specified, which will be invalidated automatically if it exceeds the specified time period.

## 4. Key management scheme

In the anti-quantum key management method for clustered sensor networks proposed in this scheme, the trusted third-party security host generates the system security parameters needed for the key management scheme. then the identity and public-private key pairs of each cluster head sensor node are generated by these security parameters, and the privacy information is pre-distributed to the corresponding cluster head sensor nodes. The cluster head sensor node plays a key role in the network, collecting the information sent by the ordinary sensor node in the cluster and forwarding it to the host. The sensor nodes in each cluster communicate securely through the symmetric key shared in the cluster, and the ordinary sensor nodes in the cluster can communicate directly. Indirect communication can be carried out through the cluster head and the sensor nodes outside the cluster. When both sides of the communication feel threatened, the cluster head sensor node can also communicate directly by negotiating the communication key. In order to further improve the security of the symmetric key used for communication, the scheme proposed in this paper stipulates that if the specified period of time is exceeded, the symmetric key will automatically expire and the new communication key will be redistributed through the host.

The key management scheme involves a few parameters: a prime *q*_1_ and prime *q* modulus, *q*,*q*_1_≥2 and integer dimensions *n*,*k*,*d*,*τ*,*λ*≥1. The key management scheme will be generated as shown in the following:

### 4.1. Sensor node identity and key distribution

1. Distribution of public and private key pairs, communication keys and identification of *Host* (Host) and cluster head sensor nodes
  1. Choose a random public matrix $A\in Z_{q}^{n\times m}$.
  2. Choose random parameter x: *x*_*i*_←{−*τ*,⋯,*τ*}^*n*^, *i* = 1,2,⋯,*Q*,*h*, where i, h and Q represents the i-th cluster head sensor node, h represents the *Host* and total number of cluster head sensor node respectively.
  3. Compute: $y_{i}^{T}=x_{i}^{T}\cdot A\mathrm{mod}q$, *i* = 1,2,⋯,*Q*,*h*.
  4. The public key of the *Host* is (*A*,*y*_*h*_), the private key of the *Host* is *x*_*h*_, identification of the *Host* is *ID*_*h*_.
  5. The public key of the i-th cluster head sensor node is (*A*,*y*_*i*_), the private key of of the i-th cluster head sensor node is *x*_*i*_.
  6. The identification of *C*^*i*^ (the i-th cluster head sensor node) is *ID*_*i*_, where $ID_{i}\in {\left\{0,1\right\}}^{n},i=1,2,\cdots ,Q$.
  7. The communication key of each cluster head sensor node is *k*_*i*_, *i* = 1,2,⋯,*Q*, which is the symmetric key of some traditional symmetric cryptosystem (RSA encryption system, etc.).
2. Distribution of public and private key pairs, communication keys and identification of ordinary sensor nodes

1. The identification of $C_{j}^{i}$ (the i-th ordinary sensor node) is *ID*_*i*_, $ID_{j}^{i}\in {\left\{0,1\right\}}^{n},i=1,2,\cdots ,Q,j=1,2,\cdots ,Q_{i}$, where, i represents that the ordinary sensor node belongs to the i-th cluster, j represents that the ordinary sensor node is the j-th sensor node in the i-th t cluster, and *Q*_*i*_ represents the total number of ordinary sensor nodes in the i-th cluster.
2. The communication key of the ordinary sensor node in each cluster is *k*_*i*_, *i* = 1,2,⋯,*Q*, where, i represents the i-th cluster.
3. The public key of the ordinary sensor node in each cluster is (*A*,*y*_*i*_), which is the public key of the i-th cluster head sensor node.

### 4.2. Communication key invalidation

Assuming that the communication key of the *i*-th cluster is a failure (including various cases such as theft, etc.), the *i*-th cluster head sensor node immediately broadcasts a communication key failure information to all sensor nodes in the *i*-th cluster, and re-authenticates and negotiates the new communication key with the host. Finally, the negotiated new communication key is peer-to-peer sent to the valid sensor node in the *i*-th cluster. The specific process of renegotiating the new communication key with:

1) First of all, it is necessary to carry out mutual authentication, the authentication process of *Host* to *C*^*i*^

***The i-th cluster head sensor node C***^***i***^:

1. Input the public key (*A*,*y*_*i*_), the private key *x*_*i*_, and identification *ID*_*i*_.
2. Choose random parameter: *θ*_*i*_←{−*τ*,⋯,*τ*}^*m*^.
3. Compute: $\eta =\theta_{i}^{T}\cdot x_{i}^{T}\cdot q_{1}+ID_{i}^{T}\mathrm{mod}q$
4. Choose a hash function *H*:
$$H:{\left\{0,1\right\}}^{*}\to \{v_{1}:v_{1}\in {\left\{0,1\right\}}^{l}\}.$$
5. Compute: $\mu_{i}=H(ID_{i}^{T}\cdot A)$.
6. Compute: $\eta^{'}=\theta^{T}\cdot y_{i}^{T}\cdot q_{1}\mathrm{mod}q$.
7. Output the signature message (*η*,*η*’,*μ*_*i*_), and *C*^*i*^ send the signed message (*η*,*η*’,*μ*_*i*_) to *Host*. Otherwise, return to regenerate the signature.

***The verification process of Host***:

1. Input the public key (*A*,*y*_*i*_), the signature message (*η*,*η*’,*μ*_*i*_), and the identification *ID*_*i*_.
2. Compute: $\mu_{i}^{'}=H(\eta \cdot A-\eta^{'}\mathrm{mod}q)$.
3. Verification: $\mu_{i}^{'}\overset{?}{=}\mu_{i}$.

If the above equation is true and the authentication process of *Host* to *C*^*i*^ is successful, proceed to the next step, otherwise terminate the key negotiation process.

2) The authentication process of *C*^*i*^ to *Host*

***The host Host***:

1. Input the public key (*A*,*y*_*h*_), the private key *x*_*h*_, and identification *ID*_*h*_.
2. Choose random parameter: *θ*_*h*_←{−*τ*,⋯,*τ*}^*m*^.
3. Compute: $\eta_{h}=\theta_{h}^{T}\cdot x_{h}^{T}\cdot q_{1}+ID_{h}^{T}\mathrm{mod}q$
4. Compute: $\mu_{h}=H(ID_{h}^{T}\cdot A)$.
5. Compute: $\eta_{h}^{'}=\theta_{h}^{T}\cdot y_{h}^{T}\cdot q_{1}\mathrm{mod}q$.
6. Output the signature message $\left(\eta_{h},\eta_{h}^{'},\mu_{h}\right)$, and *Host* send the signed message $\left(\eta_{h},\eta_{h}^{'},\mu_{h}\right)$ to *C*^*i*^. Otherwise, return to regenerate the signature.

***The verification process of C***^***i***^:

1. Input the public key (*A*,*y*_*h*_), the signature message $\left(\eta_{h},\eta_{h}^{'},\mu_{h}\right)$, and the identification *ID*_*h*_.
2. Compute: $\mu_{h}^{'}=H(\eta_{h}\cdot A-\eta_{h}^{'}\mathrm{mod}q)$.
3. Verification: $\mu_{h}^{'}\overset{?}{=}\mu_{h}$.

If the above equation is true and the authentication process of *Host* to *C*^*i*^ is successful, proceed to the next step, otherwise terminate the key negotiation process.

3) The *Host* reassigns a new communication key

The *Host* generates a new communication key $k_{i}^{'}$, encrypts $k_{i}^{'}$ with its own private key *x*_*h*_, and then sends the encrypted message $\left(m_{k},m_{k}^{'},k_{H}\right)$ to the *i*-th cluster head sensor node *C*^*i*^. After receiving the message from the host, *C*^*i*^ decrypts the message $\left(m_{k},m_{k}^{'},k_{H}\right)$ with the public key (*A*,*y*_*h*_) of the *Host*. After decryption, the *i*-th cluster head sensor node *C*^*i*^ obtains a new communication key $k_{i}^{'}$, and the *i*-th cluster head sensor node can securely communicate with the *Host* with the new communication key $k_{i}^{'}$. The specific process is as follows:

***The encryption process of Host***:

1. Input the public key (*A*,*y*_*i*_) of *C*^*i*^ and the new communication key $k_{i}^{'}$.
2. Choose random parameters: *θ*_*k*_←{−*τ*,⋯,*τ*}^*m*^, *e*←{−*τ*,⋯,*τ*}^*n*^, *e*’←{−*τ*,⋯,*τ*}.
3. Compute: $m_{k}=q_{1}\cdot A\cdot \theta_{k}+q_{1}\cdot e\mathrm{mod}q$.
4. Choose a random message $k_{i}^{'}$: $k_{i}^{'}\in {\left\{0,1\right\}}^{*}$.
5. Compute: $k_{H}=H(k_{i}^{'})$.
6. Compute: $m_{k}^{'}=q_{1}\cdot y_{i}^{T}\cdot \theta_{k}+q_{1}\cdot e^{'}+k_{i}^{'}\mathrm{mod}q$.
7. Output the ciphertext message $\left(m_{k},m_{k}^{'},k_{H}\right)$, and *Host* send the signed message $\left(m_{k},m_{k}^{'},k_{H}\right)$ to *C*^*i*^.

***The decryption process of C***^***i***^:

1. Input the private key $x_{i}$, the ciphertext message $\left(m_{k},m_{k}^{'},k_{H}\right)$.
2. Compute: $k_{H}^{'}=H((m_{k}^{'}-x_{i}^{T}\cdot m_{k}\mathrm{mod}q)\mathrm{mod}q_{1})$.
3. Verification: $k_{H}^{'}\overset{?}{=}k_{H}$.

If the above equation is true and the decryption process is successful, *C*^*i*^ get a new communication key $k_{i}^{'}$: $k_{i}^{'}=(m_{k}^{'}-x_{i}^{T}\cdot m_{k}\mathrm{mod}q)\mathrm{mod}q_{1}$. Otherwise, decryption fails.

4) ***C***^***i***^ **assign a new communication key for the *i*-th cluster**

The *i*-th cluster head sensor node encrypts the new communication key $k_{i}^{'}$ with its own private key *x*_*i*_: and then sends the ciphertext message $\left(m_{i},m_{i}^{'},k_{i}\right)$ point to point to all valid ordinary sensor nodes of the *i*-th cluster. After receiving the message from the *i*-th cluster head sensor node, the ordinary sensor node of the *i*-th cluster decrypts the ciphertext message $\left(m_{i},m_{i}^{'},k_{i}\right)$ with the public key of the *i*-th cluster head sensor node. After decryption, the ordinary sensor node of the *i*-th cluster obtains a new communication key $k_{i}^{'}$, and the specific process of encryption and decryption has referred to the process of *Host* reassigns a new communication key. Finally, the new communication key $k_{i}^{'}$ can be used to communicate securely with all sensor nodes (including cluster head nodes) of the *i*-th cluster.

### 4.3. Cluster head node key negotiation process

The communication key is not distributed between the cluster heads, because in general, the cluster heads do not communicate directly. If the cluster head sensor nodes in special cases must communicate directly, they can first authenticate each other and negotiate the communication key between each other. Suppose that the *i*-th cluster head sensor node and the *j*-th cluster head sensor node needs to communicate directly, and the negotiation process is as follows:

1. The *i*-th cluster head sensor node and the *j*-th cluster head sensor node authenticate each other, and the mutual authentication process is referred to the first two steps of communication key invalidation steps. If the authentication is successful, proceed to the next step, otherwise the key negotiation process is terminated.
2. The key agreement process between the *i*-th cluster head sensor node and the *j*-th cluster head sensor node the last step of communication key invalidation steps.
3. Finally, the *i*-th cluster head sensor node and the *j*-th cluster head sensor node obtains the communication key *k*_*i*⇔*j*_, through the process of mutual authentication and negotiation, and the *i*-th cluster head sensor node and the *j*-th cluster head sensor node can communicate securely with the communication key *k*_*i*⇔*j*_ directly.

## 5. Analysis

### 5.1. Correctness

The correctness of the decryption in the key management scheme follows from our choice of parameters. Specifically, to show correctness, we follow the proof strategy from [32], we first compute $k_{i}^{'}=(m_{k}^{'}-x_{i}^{T}\cdot m_{k}\mathrm{mod}q)\mathrm{mod}q_{1}$. We have:

$$k_{i}^{'}=(m_{k}^{'}-x_{i}^{T}\cdot m_{k}\mathrm{mod}q)\mathrm{mod}q_{1}$$

$$=((q_{1}\cdot y_{i}^{T}\cdot \theta_{k}+q_{1}\cdot e^{'}+k_{i}^{'}-x_{i}^{T}\cdot (q_{1}\cdot A\cdot \theta_{k}+q_{1}\cdot e))\mathrm{mod}q)\mathrm{mod}q_{1}$$

$$=((q_{1}\cdot y_{i}^{T}\cdot \theta_{k}-q_{1}\cdot x_{j}^{T}\cdot A\cdot \theta_{k}+q_{1}(e^{'}+x_{i}^{T}\cdot e)+k_{i}^{'})\mathrm{mod}q)\mathrm{mod}q_{1}$$

$$=((q_{1}\cdot (e^{'}+x_{i}^{T}\cdot e)+k_{i}^{'})\mathrm{mod}q)\mathrm{mod}q_{1}$$

Since we assumed $(n\cdot d\cdot \tau +1)\leq \frac{q}{2q_{1}}-\frac{1}{2}$ and ${\left\|k_{i}^{'}\right\|}_{\infty }\leq q_{1}/2$, then ${\left\|q_{1}\cdot (e^{'}+x_{i}^{T}\cdot e)+k_{i}^{'}\right\|}_{\infty }\leq q_{1}/2$, therefore there is no reduction modulo *q*_1_ in $q_{1}\cdot (e^{'}+x_{i}^{T}\cdot e)+k_{i}^{'}$ and hence

$$k_{i}^{'}=((q_{1}\cdot (e^{'}+x_{i}^{T}\cdot e)+k_{i}^{'})\mathrm{mod}q)\mathrm{mod}q_{1}=k_{i}^{'}.$$

Then

$$k_{H}^{'}=H(k_{i}^{'})=k_{H}$$

The correctness of the signature in the scheme follows from our choice of parameters. Specifically, to show correctness, we first compute $\mu_{H}=\eta \cdot A-\eta^{'}\mathrm{mod}q$. We have:

$$\mu_{H}=\eta \cdot A-\eta^{'}\mathrm{mod}q$$

$$=((\theta_{i}^{T}\cdot x_{i}^{T}\cdot q_{1}+ID_{i}^{T})\cdot A-\theta^{T}\cdot y_{i}^{T}\cdot q_{1})\mathrm{mod}q$$

$$=(\theta_{i}^{T}\cdot x_{i}^{T}\cdot A\cdot q_{1}+ID_{i}^{T}\cdot A-\theta^{T}\cdot y_{i}^{T}\cdot q_{1})\mathrm{mod}q$$

$$=(\theta^{T}\cdot y_{i}^{T}\cdot q_{1}+ID_{i}^{T}\cdot A-\theta^{T}\cdot y_{i}^{T}\cdot q_{1})\mathrm{mod}q$$

$$=ID_{i}^{T}\cdot A\mathrm{mod}q$$

Hence

$$\mu_{i}^{'}=H(\eta \cdot A-\eta^{'}\mathrm{mod}q)=H(\mu_{H})=H(ID_{i}^{T}\cdot A\mathrm{mod}q)=\mu_{i}.$$

### 5.2. Security

***Unforgettability***: a successful interaction between the signer and the user can only generate a legitimate signature. Here, it is proved that if there is an adversary A with the ability to resist unforgeable attacks, then the *MLWE* difficult problem can be solved in the polynomial time algorithm. That is, assuming that there is an adversary A who can successfully forge a valid message signature with a non-negligible probability *δ*, then a valid solution to the *MLWE* difficult problem can be found in polynomial time:

***Proof*:** first of all, it is emphasized that the output of the proposed signature authentication scheme is independent of the signature key. For the two main output hashes in the scheme and the signature of the message to be signed, the adversary A queries the two algorithms. Once the opponent has the ability to resist unforgeable attacks, the challenger *T* will be able to solve the *MLWE* difficult problems.

***Hash query*:** the challenger *T* creates an initially empty list *L*_*H*_ to store the hash query value for the message $ID_{i}^{T}\cdot A\mathrm{mod}q$. When the challenger *T* receives a hash query about the message from the adversary A, the challenger *T* first checks the list *L*_*H*_ to see if the message has been queried. If queried, the message and hash result pair $\left(ID_{i}^{T}\cdot A\mathrm{mod}q,H(ID_{i}^{T}\cdot A\mathrm{mod}q)\right)$ is sent to the adversary A, otherwise, the challenger *T* runs the algorithm to regenerate the hash value $\left(ID_{i}^{T}\cdot A\mathrm{mod}q,H(ID_{i}^{T}\cdot A\mathrm{mod}q)\right)$ of a message $ID_{i}^{T}\cdot A\mathrm{mod}q$, sends the result to the adversary A, and stores the message and hash result pair $\left(ID_{i}^{T}\cdot A\mathrm{mod}q,H(ID_{i}^{T}\cdot A\mathrm{mod}q)\right)$ in the list *L*_*H*_.

1. Choose random parameters: *θ*_*i*_←{−*τ*,⋯,*τ*}^*m*^.
2. Compute: $\eta =\theta_{i}^{T}\cdot x_{i}^{T}\cdot q_{1}+ID_{i}^{T}\mathrm{mod}q$.
3. Compute: $\mu_{i}=H(ID_{i}^{T}\cdot A)$.
4. Compute: $\eta^{'}=\theta^{T}\cdot y_{i}^{T}\cdot q_{1}\mathrm{mod}q$.
5. Compute: $\mu_{H}=\eta \cdot A-\eta^{'}\mathrm{mod}q$.
6. Compute: $\mu_{i}^{'}=H(\mu_{H})$.
7. Verification: $\mu_{i}^{'}\overset{?}{=}\mu_{i}$.

***Forgery*:** suppose *μ*_*H*,*j*_ is the result of a hash query returned to the adversary A, which can be obtained for two different signature pairs $\left(\eta ,\eta^{'},\mu_{H,j}\right)$ and $\left(\eta^{*},\eta^{'*},\mu_{H,j}^{*}\right)$, then $H(\eta \cdot A-\eta^{'}\mathrm{mod}q)=H(\eta^{*}\cdot A-\eta^{'*}\mathrm{mod}q)$. Because *η* ≠ η* and $\eta \cdot A-\eta^{'}\neq \eta^{*}\cdot A-\eta^{'*}$, there will have a hash collision. But the hash collision can hardly happen because of the collision resistance of the hash function. Therefore, it can be obtained with a higher probability *η* = η* and $\eta \cdot A-\eta^{'}=\eta^{*}\cdot A-\eta^{'*}$, then, $\mu_{H,j}=\mu_{H,j}^{*}$. Finally, it can be claimed that the *MLWE* difficult problem has been successfully solved, the detailed process is as follows:

The *μ*_*H*,*j*_ is assumed that the challenger returns the result of the hash query to the adversary A. For the signature of the message $\left(\eta^{*},\eta^{'*},\mu_{H}^{*}\right)$, select different random values $\mu_{H,j}^{*},\mu_{H,2}^{*},\cdots ,\mu_{H,s}^{*}\leftarrow D_{\kappa }^{n}$. The probability of $\mu_{H,j}\neq \mu_{H,j}^{*}$ can be obtained:

$$\mathrm{Pr}(\mu_{H,j}\neq \mu_{H,j}^{*})=(\delta -1/D_{\kappa }^{n})\times (\frac{\delta -1/D_{\kappa }^{n}}{t}-1/D_{\kappa }^{n})$$

Therefore, the adversary A can forge a new signature $\left(\eta^{*},\eta^{'*},\mu_{H,j}^{*}\right)$ and $\eta \cdot A-\eta^{'}=\eta^{*}\cdot A-\eta^{'*}$ according to the system parameter setting of the signature authentication scheme, the following equation can be obtained:

$$(\eta^{'}-\eta^{'*})\mathrm{mod}q=(\eta -\eta^{*})\cdot A\mathrm{mod}q.$$

Due to $(\eta^{'}-\eta^{'*})\mathrm{mod}q=(\eta -\eta^{*})\cdot A\mathrm{mod}q$, then:

$$\mu_{i}=H(\eta \cdot A-\eta^{'}\mathrm{mod}q)=H(\eta^{*}\cdot A-\eta^{'*}\mathrm{mod}q)=\mu_{i}^{'*}.$$

Next, $(n\cdot d\cdot \tau +1)\leq \frac{q}{2q_{1}}-\frac{1}{2}$ and ${\left\|k_{i}^{'}\right\|}_{\infty }\leq q_{1}/2$ can be obtained with a non-negligible probability, that is, a solution of the *MLWE* difficult problem is solved in the polynomial time.

However, because the *MLWE* difficult problem can’t be solved in polynomial time, the assumption of adversary A is not valid. Therefore, the proposed signature authentication of key management scheme satisfies the unforgeability in the random prophecy model.

### 5.3. Efficiency analysis

In this sec tion, we mainly focus on the algorithm computational complexity between our lattice-based key management protocol and other related secret key protocols, ref. [4] protocol, ref. [15] protocol and ref. [20] protocol. The test environment of this scheme is that the Intel Core i7-12700 processor is configured with 32G-DDR4 memory, the operating system is Windows10; test programming language is Python3.9, and the code function is implemented by PyCryptodome library. The results are shown in Table 1.

**Table 1:** Comparison with RSA and ECC algorithms.

Security level	RSA algorithm	ECC algorithm	Our algorithm
64 B	4.163KB	1.056KB	59.893KB
128B	8.326KB	2.112KB	61.072KB
192 B	12.489KB	3.168KB	62.557KB
256B	16.652KB	4.224KB	63.981KB
320B	20.815KB	5.028KB	65.026KB
384B	24.978KB	6.336KB	66.133KB
448B	29.141KB	7.392KB	67.317KB
512B	33.304KB	8.448KB	68.587KB

According to the above analysis, the message authentication size of the proposed authentication protocol is *m*log(12*σ*), which is only related to the message *m* and the parameter *σ*. The authentication sizes corresponding to different security levels (such as 64bits, 128bits, 192bits, 256bits, 320bits, 384bits,448bits and 512bits) can be calculated when the selected system parameter is *n* = 256,*q* = 2^32^. The results are shown in Table 1. The algorithm authentication size corresponding to different security levels of our proposed lattice-based cipher scheme and RSA and ECC authentication algorithms is given. As shown in Table 1, with the continuous improvement of the security level of the RSA algorithm, the required authentication size increases very quickly, which is not suitable for encrypting large data in high-level security. However, the size of the lattice-based authentication proposed in this paper does not change much. The size of the authentication is kept at a stable level, which is more suitable for encrypting large data in high-level security. The size of the certification of the ECC algorithm grows slightly slower than that of the RSA algorithm, but its certification also doubles as the security level of the algorithm increases. In addition, the schemes implemented with RSA and ECC algorithms cannot resist quantum computing attacks, so the lattice-based authentication protocol in this paper has good anti-quantum security. With the development of quantum computers and quantum computing, lattice cryptography will be a very practical cryptographic algorithm in the quantum era.

Therefore, the lattice-based scheme proposed in this paper has better security, and when the security level is higher, the algorithm efficiency has certain advantages.

## 6. Conclusions

Utilizing the cluster management of wireless sensor networks, most sensor nodes only need a small amount of storage space, effectively reducing deployment costs and reducing the number of mutual authentication between sensor nodes, which is suitable for medium and large-scale deployment of sensor networks. After mutual authentication, the sensor nodes in the wireless sensor network use symmetric keys for data communication. Since the amount of data that needs to be communicated is much greater than the amount of data that needs to be authenticated, the data communication is carried out through the traditional cryptographic system, which effectively improves the data security and communication efficiency. The cluster sensor network key management method proposed in this paper has the advantages of simple process, high security and high efficiency. The use of cluster structure can reduce the cost of frequent mutual authentication brought by transmission, which is beneficial to the expansion of the network. It is suitable for deployment in applications such as forest fire prevention and urban air quality monitoring. Even in the post-quantum era, it can well guarantee the security of mutual authentication between sensor nodes, and has broad practical application prospects. The size of the lattice-based authentication proposed in this paper does not change much with the continuous improvement of the security level of the RSA algorithm. The size of the certificate is kept at a stable level, which is more suitable for encrypting large data at a high security level.

For future work, we will continue to investigate lattice-based quantum computing-resistant key management schemes that support more flexible signature strategies.

## References

[1] Kizilkaya B., Ever E., Yatbaz H. Y., and Yazici A., “An Effective Forest Fire Detection Framework Using Heterogeneous Wireless Multimedia Sensor Networks,” *ACM Trans*. *Multimedia Comput*. *Commun*. *Appl*., vol.18, no. 2, pp.1–21, May 2022.

[2] Tang J, Lu X, Xiang Y, Shi C, Gu J. Blockchain search engine: Its current research status and future prospect in Internet of Things network. Future Generation Computer Systems. 2023, 138(1):120–141.

[3] Priyadarshi R. and Gupta B., “Area Coverage Optimization in Three-Dimensional Wireless Sensor Network,” *Wireless Personal Communications*, vol.117, no. 2, pp.843–865, 2021.

[4] Maller Mary, Bowe Sean, Kohlweiss Markulf, and Meiklejohn Sarah, “Sonic: Zero-knowledge snarks from linear-size universal and updatable structured reference strings,” in Proceedings of the 2019 ACM SIGSAC Conference on Computer and Communications Security, vol. 2019, pp. 2111–2128, 2019.

[5] Khot P. S. and Naik U. L., “Cellular automata-based optimised routing for secure data transmission in wireless sensor networks,” *Journal of Experimental & Theoretical Artificial Intelligence*, vol.34, no. 3, pp.431–449, May 2022.

[6] Al Nuaimi MMK, Rishal KP, Oommen NV, Sherimon PC. Blockchain Implementation Framework for Tracing the Dairy Supply Chain. Lecture Notes on Data Engineering and Communications Technologies. 2023, 142(1):551–560.

[7] Tuan N. A., Akila D., Pal S., Sarkar B., Khai Tran T., Mothilal Nehru G., et al., “Dynamic Data Optimization in IoT-Assisted Sensor Networks on Cloud Platform,” *Computers*, *Materials & Continua*, vol.72, no. 1, pp.1357–1372, 2022.

[8] Nain M. and Goyal N., “Energy Efficient Localization Through Node Mobility and Propagation Delay Prediction in Underwater Wireless Sensor Network,” *Wireless Personal Communications*, no. 2, pp.1–19, 2021.33558792

[9] Zhang J, Li T, Obaidat M S, et al. Enabling efficient data sharing with auditable user revocation for 10V systems. IEEE Systems Journal, 2022, 16(1): 1355–1366.

[10] Mehmood A., Umar M. M., and Song H., “ICMDS: Secure inter-cluster multiple-key distribution scheme for wireless sensor networks,” *Ad Hoc Networks*, vol.55, no. 6, pp.97–106, 2016.

[11] Zhang J., Li H., and Li J., “Key Establishment Scheme for Wireless Sensor Networks Based on Polynomial and Random Key Predistribution Scheme,” *Ad Hoc Networks*, vol.71, no. MAR., pp.68–77, 2017.

[12] Kumar D., Pachigolla S. K., Manhas S. S., and Rawat K., “Cryptanalysis and improvement of mutual authentication protocol for real-time data access in industrial wireless sensor networks,” *International Journal of Computers and Applications*, vol.44, no. 6, pp.521–534, Jun. 2022.

[13] Palani U., Amuthavalli G., and Alamelumangai V., “Secure and load balanced routing protocol in wireless sensor network for disaster management,” *IET Information Security*, vol.14, no. 5, pp.513–520, 2020.

[14] D’anvers JP, Van Beirendonck M, Verbauwhede I. Revisiting Higher-Order Masked Comparison for Lattice-Based Cryptography: Algorithms and Bit-Sliced Implementations. IEEE Transactions on Computers. 2023, 72(2):321–332.

[15] Benedikt Bünz Jonathan Bootle, Boneh Dan, Poelstra Andrew, Wuille Pieter, and Maxwell Greg, “Bulletproofs: Short proofs for confidential transactions and more,” in 2018 IEEE symposium on security and privacy (SP), pp. 315–334. IEEE, 2018.

[16] Li H., Guo F., Wang L., Wang J., Wang B., and Wu C., “A Blockchain-Based Public Auditing Protocol with Self-Certified Public Keys for Cloud Data,” *Security and Communication Networks*, vol.2021, pp.1–10, Feb. 2021.

[17] Tahat N., Alomari A. K., Al-Hazaimeh O. M., and Al-Jamal M. F., “An efficient self-certified multi-proxy signature scheme based on elliptic curve discrete logarithm problem,” *Journal of Discrete Mathematical Sciences and Cryptography*, vol.23, no. 4, pp.935–948, May 2020. doi: 10.1080/09720529.2020.1734293

[18] Yamamura K, Wang Y, Fujisaki E. Improved lattice enumeration algorithms by primal and dual reordering methods. IET Information Security. 2023, 17(1):35–45.

[19] Islam N, Marinakis Y, Olson S, White R, Walsh S. Is BlockChain Mining Profitable in the Long Run. IEEE Transactions on Engineering Management. 2023, 70(2):386–399.

[20] Eli Ben-Sasson Iddo Bentov, Horesh Yinon, and Riabzev Michael, “Scalable, transparent, and postquantum secure computational integrity,” Cryptology ePrint Archive, vol. 2018, pp. 46–128, 2018.

[21] Zhang J., Cui J., Zhong H., Chen Z., and Liu L., “PA-CRT: Chinese Remainder Theorem Based Conditional Privacy-Preserving Authentication Scheme in Vehicular Ad-Hoc Networks,” *IEEE Trans*. *Dependable and Secure Comput*., vol.18, no. 2, pp.722–735, Mar. 2021.

[22] Ametepe A. F.-X., Ahouandjinou A. S. R. M., and Ezin E. C., “Robust encryption method based on AES-CBC using elliptic curves Diffie–Hellman to secure data in wireless sensor networks,” *Wireless Netw*, vol.28, no. 3, pp.991–1001, Apr. 2022. doi: 10.1007/s11276-022-02903-3

[23] Mehmood G, "An efficient and secure session key establishment scheme for health-care applications in wireless body area networks," *J*. *Eng*. *Appl*., 2018, pp:1–6.

[24] Bootle J., Lyubashevsky V., and Seiler G., “Algebraic Techniques for Short(er) Exact Lattice-Based Zero-Knowledge Proofs,” *in Advances in Cryptology–CRYPTO 2019*, vol.11692, pp.176–202, 2019.

[25] Mehmood G, Khan M Z, Waheed A, Zareei M and Mohamed E M, "A trust-based energy-efficient and reliable communication scheme (trust-based ercs) for remote patient monitoring in wireless body area networks," *IEEE Access*, 2020, pp:1–9.

[26] Lyubashevsky V., Nguyen N. K., and Seiler G., “Shorter Lattice-Based Zero-Knowledge Proofs via One-Time Commitments,” *in Public-Key Cryptography–PKC 2021*, vol.12710, pp.215–241, 2021.

[27] Mehmood G, Khan M S, Waheed A, Zareei M, Fayaz M, and Sadad T, "An efficient and secure session key management scheme in wireless sensor network," *Complexity*, 2021, pp:1–6.

[28] Mehmood G, Khan M S, Fayaz M, Faisal M, Rahman H U, and Gwak J, "An energy-efficient mobile agent-based data aggregation scheme for wireless body area networks," *Computers*, *Materials & Continua*, 2022, vol.70, no.3, pp:5929–5948.

[29] Dharminder D, Reddy CB, Das AK, Park Y, Jamal SS. Post-Quantum Lattice-Based Secure Reconciliation Enabled Key Agreement Protocol for IoT. IEEE Internet of Things Journal10. 2023, 10(3):2680–2692.

[30] Lyubashevsky V., “Lattice Signatures without Trapdoors,” *in Advances in Cryptology–EUROCRYPT 2012*, vol.7237, pp.738–755, 2012.

[31] Langlois A. and Stehlé D., “Worst-case to average-case reductions for module lattices,” *Des*. *Codes Cryptogr*., vol.75, no. 3, pp.565–599, Jun. 2015.

[32] Lyubashevsky V., Nguyen N. K., and Plancon M., “Efficient Lattice-Based Blind Signatures via Gaussian One-Time Signatures,” *in Public-Key Cryptography–PKC 2022*, vol.13178, pp.498–527, 2022.

[33] Corentin J., Adeline R. L. and Olivier S., Lattice-Based Signature with Efficient Protocols, Revisited, *eprint*.*iacr*.*org*, 1–46, 2022, https://eprint.iacr.org/2022/509.

